# Optimum Configuration of Cannulated Compression Screws for the Fixation of Unstable Femoral Neck Fractures: Finite Element Analysis Evaluation

**DOI:** 10.1155/2018/1271762

**Published:** 2018-12-09

**Authors:** Jiantao Li, Menglin Wang, Jianfeng Zhou, Lin Han, Hao Zhang, Chen Li, Lianting Li, Ming Hao

**Affiliations:** ^1^Department of Orthopaedics, Chinese PLA General Hospital, No. 28 Fuxing Road, Beijing 100853, China; ^2^Department of Otolaryngology Head and Neck Surgery, Peking University Third Hospital, Beijing 100191, China; ^3^Department of Emergency, Chinese PLA General Hospital, No. 28 Fuxing Road, Beijing 100853, China; ^4^Graduate School of the Second Military Medical University, Shanghai 200433, China; ^5^Department of Orthopaedics, Tianjin Hospital, No. 406 Jiefang Road, Tianjin 300211, China; ^6^Department of Orthopaedics, The Third People's Hospital of Qingdao, No. 29 Yongping Road, Qingdao 266041, China

## Abstract

**Objectives:**

In the present study, we evaluated the mechanical outcome of different configurations of cannulated compression screws for the fixation of Pauwels type III femoral neck fracture and the stress distribution around the holes corresponding to fixation protocol after screws removal.

**Methods:**

The Pauwels type III of femoral neck fracture was created in 3-matic software and the models of cannulated compression screws were constructed using UG-NX software. Five fixation systems were assembled to the fracture models. Abaqus software was used to perform the process of finite element analysis. Values of stress distribution, maximum stress, model principal strains of proximal fragment, and stress distribution around the holes of femur model were recorded.

**Results:**

Stress of cannulated compression screws was intensely focused on the middle area of the screw near the fragment of each group. Inverted triangle model showed the highest peak stress on screws under different phases of load. Each screw dispersed some stresses, but at least one underwent the peak stress. Fracture model fixed by inverted triangle configuration showed the lowest volume of yielding strain in the proximal fragment. The area of higher stress around the holes was largest after triangle screws removal when compared with other four models.

**Conclusions:**

Our study indicated that different cannulated compression screws fixation configurations for the unstable femoral neck fractures showed the different mechanical efficiency. Inverted triangular configuration showed the mechanical advantage and being less likely to cutout. The fixation strategy of triangle configuration was least recommended if patients tended to remove the implants.

## 1. Introduction

Femoral neck fracture (FNF) is a major public health problem and a common injury encountered by orthopaedic surgeons, which accounts for about 50% of hip fractures [1]. Treatment recommendations depend on the patient's age and fracture type [2]. Various treatment strategies, including compression screws, locked plates, dynamic condylar screws, and sliding hip screws, are available to treat young patients with FNFs by anatomic reduction and stable fixation to minimize the possibility of nonunion and osteonecrosis [3–5]. Despite the method of the fixation, prevalence of complications like nonunion and fixation failure has been reported between 10 and 30% [6].

Fixation by three cannulated compression screws (CCS) has been remained a standard method for fixation of FNFs for many years [7, 8], although debate continues regarding the configurations of screws fixation. Furthermore, there is no consensus on the optimal configuration of the screws. Even if fractures have healed uneventfully, internal fixation can sometimes cause functional impairment and local irritation [9, 10]. Therefore, surgeons are asked to remove implant after fracture healing by some patients with persistent complaints. But subtrochanteric fractures and other complications related to hardware removal have been well documented [11–13]. It is unclear how the holes on the proximal femur after screws removal affect the structural integrity of the bone. Seeking to analyze the stress distribution around the holes is of significant interest and medical benefit.

To our best knowledge, few biomechanical studies of finite element analysis (FEA) test are reported to evaluate the biomechanical properties of different three-CCS configurations used in the certain unstable FNFs. Therefore, we have designed this study to compare the mechanical distinction of different CCS scenarios for the fixation of unstable FNFs. We also evaluate stress distribution around the holes corresponding to fixation protocol after screws removal.

## 2. Materials and Methods

**Figure 1:**
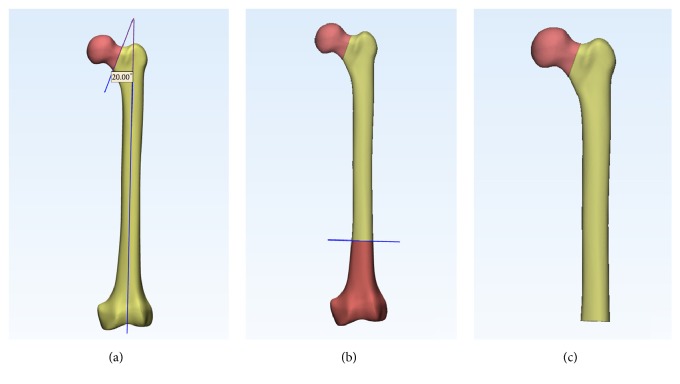
(a) Sagittal plane and cutting plane were made, respectively. (b) A distal osteotomy plane was made. (c) Pauwels type III model was created.

A geometric model of a left fourth-generation composite femur (MODEL3405#, Pacific Research Laboratories, Vashon, WA) was used in this study. Femoral neck fracture type of Pauwels III [14] was created in 3-matic software (Materialize, Belgian). The Pauwels classification is based on the angle that is formed by the fracture line and the horizontal line. Type III fractures are greater than 50 degrees. We firstly created axis of femoral shaft, through which a sagittal plane was made. The femoral neck fracture line was then created at its center by a cutting plane that was made at an angle of 20 degrees to the sagittal plane, simulating a Pauwels type III fracture (Figure 1(a)). A distal osteotomy plane was made at 10 cm above condyles (Figure 1(b)). Finally, the fracture model was created (Figure 1(c)).

**Figure 2:**
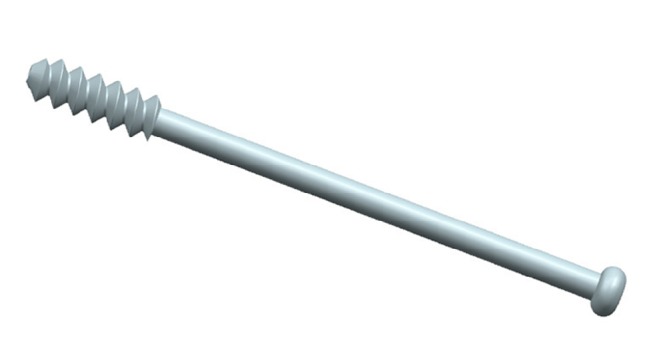
Geometric 3-D model of CCS.

**Figure 3:**
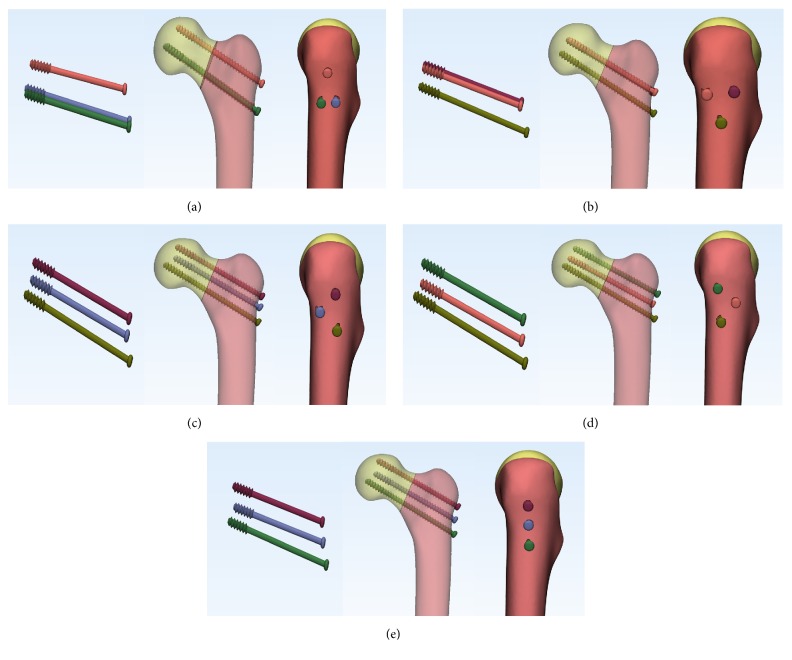
Assemblage of the CCSs and fracture models: (a) triangle, (b) inverted triangle, (c) anterior triangle, (d) posterior triangle, and (e) vertical model.

**Figure 4:**
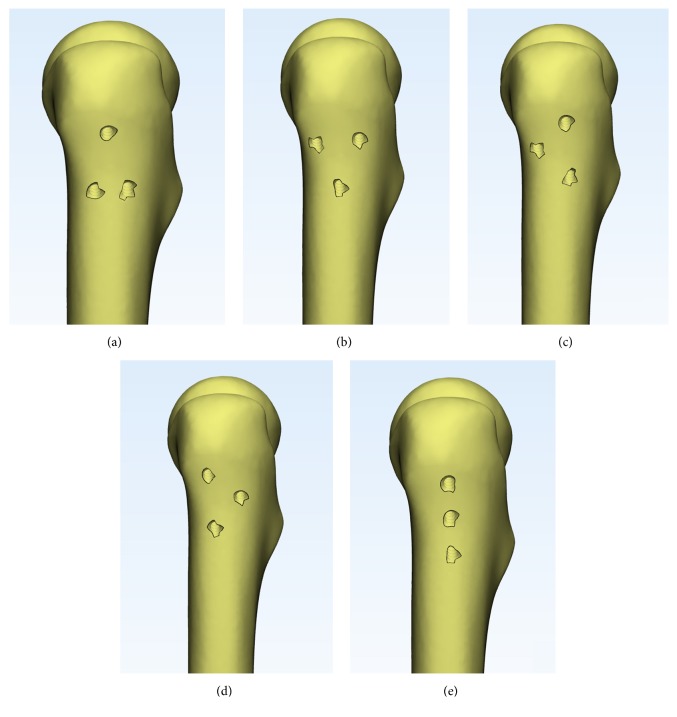
Union models after removal of the implants were simulated in 3-matic: (a) triangle, (b) inverted triangle, (c) anterior triangle, (d) posterior triangle, and (e) vertical model.

The 3-D models (Figure 2) of CCS (diameter: 6.5 mm; thread length: 16 mm) was reconstructed using the software of Unigraphics NX 8.5 (Siemens PLM Software). We completed the assemblage process of screws and bones in the 3-matic software to simulate configurations of triangle, inverted triangle, anterior triangle, posterior triangle, and vertical model (Figure 3). All the screws were perpendicular to the fracture line. Screws in each group were placed close to endosteal cortex. The inferior screw in each model was taken to avoid placing the inferior screw below the lesser trochanter level. The definition of the insertion level of CCSs could be seen in Tables 1 and 2. The threaded tunnels left by CCSs in models were also simulated using the software of 3-matic. We simulated union models after the removal of the implants (Figure 4). We meshed the models using the software of HyperMesh 11.0 (Altair Engineering, Inc., USA). The mesh was refined until the resulting displacements converged between the models. Around the screw holes, the mesh was refined.

FEA process was performed in the software of Abaqus (Simulia, France). Convergence tests were performed on all models to ensure a fine enough element discretization for stress analysis. Bone was assumed to be homogeneous, isotropic with linear elastic properties [15–18]. Details of Poisson's ratio and elasticity modulus were listed in Table 3. CCS was made of Ti-6AL-4V. Finite element models were meshed using tetrahedral 10-nodes elements (C3D10). The effect of gravity was considered as negligible in the model.

Contact interactions between bone and CCS, between bone fragments, were assumed to be frictional. The threaded surface of CCS was considered to be tie constraints. A friction factor of 0.3 was set as the interfaces between bone and CCS body and friction coefficient of 0.46 for bone-bone interaction [19]. The distal end of the femur surface was constrained with 0 degrees of freedom.

The finite element (FE) models were subjected to a load of 2100N corresponding to 300% body weight. The force vector applied to the femoral head laterally in the coronal plane at an angle of 13 degrees with femoral shaft axis. In the sagittal plane, the force vector applied posteriorly at an angle of 8 degrees with the femoral shaft axis [20]. In the FEA process, the force was divided into 4 steps, in order to simulate the gradually weight bearing postoperatively. Values of the von Mises stress distribution on the CCSs, maximum stress, model principal strains of proximal fragment, and stress distribution around the holes of femur model were recorded.

## 3. Results

Parameters of models were summarized in Table 4.

### 3.1. Von Mises Stress Distribution

**Figure 5:**
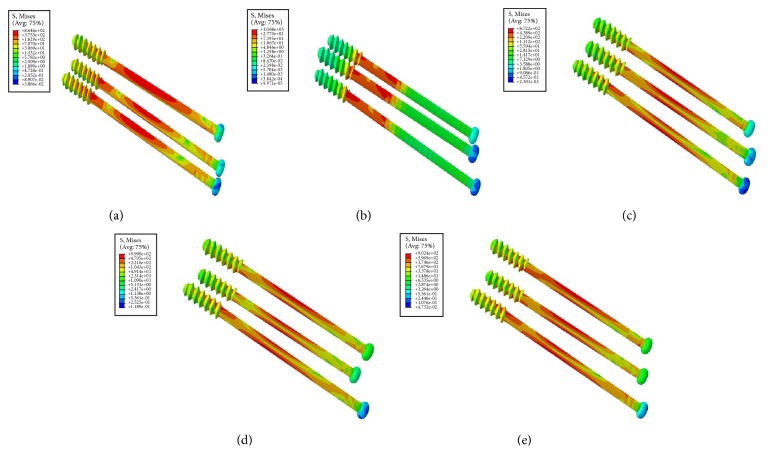
The stress nephogram of different CCS configurations: (a) triangle, (b) inverted triangle, (c) anterior triangle, (d) posterior triangle, and (e) vertical model.

**Figure 6:**
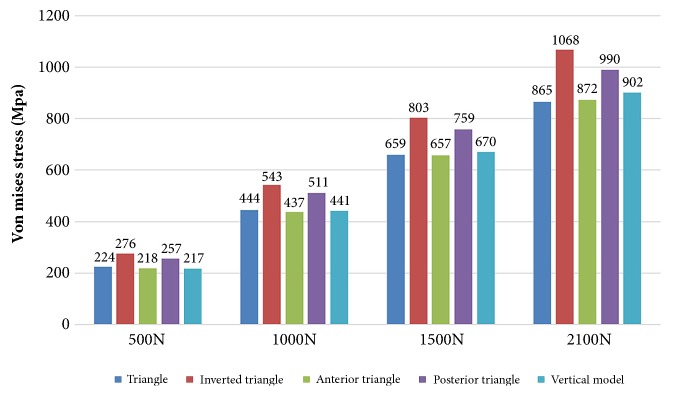
Bar chart showing the peak stress variety of five CCS configurations under different phases of loads.

**Figure 7:**
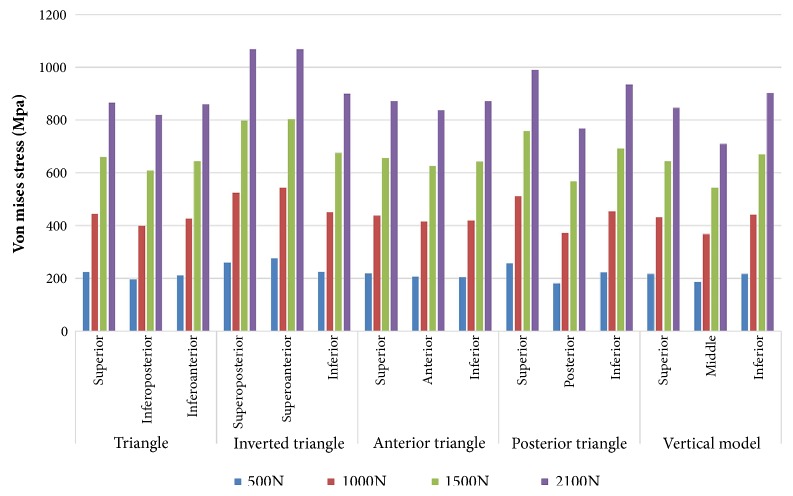
Bar chart showing the peak stress on each screw in five CCS configurations under different phases of loads.

Condition of stress distribution was shown in Figure 5. Stresses were intensely focused on the middle area of the screw near the fragment of each group. Model of inverted triangle showed the highest peak stress on CCSs under different phases of loads. Lower stress values were noticed in models of triangle and anterior triangle (Figure 6). Each screw in one model dispersed some stresses, but at least one underwent the peak stress (Table 5 and Figure 7).

### 3.2. Principal Strains in Proximal Fragment

**Figure 8:**
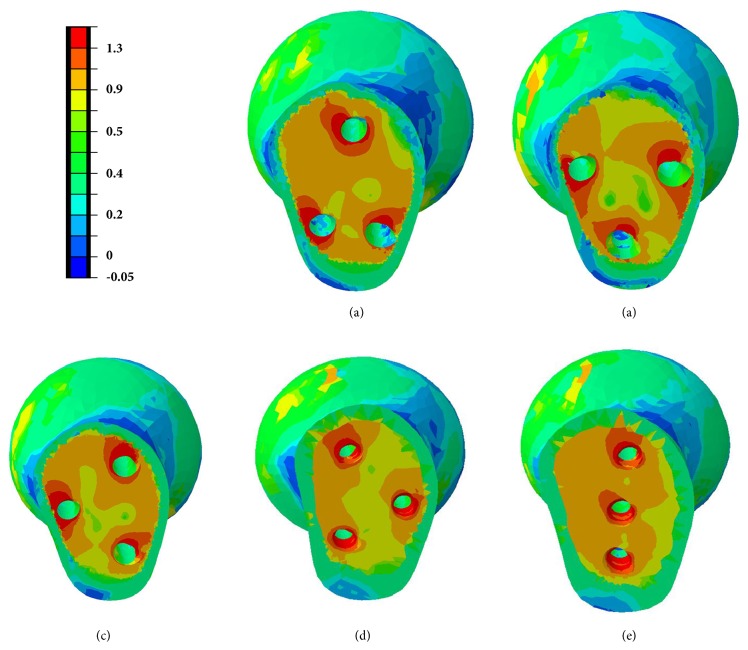
Strain nephograms showing the principal strains plotted in percent with a yield strain value of 0.9%. Orange regions indicating the strains above 0.9% and at higher risk of cutout. (a) Triangle model. (b) Inverted triangle model. (c) Anterior triangle model. (d) Posterior triangle model. (e) Vertical model.

**Figure 9:**
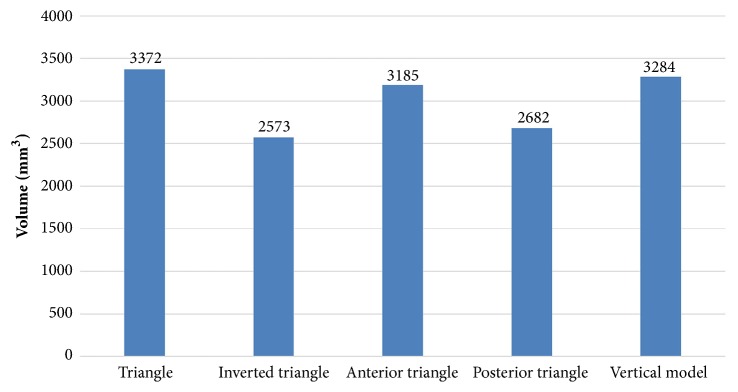
Bar chart showing the cancellous bone volume in the proximal fragment susceptible to yielding.

Strain nephograms demonstrated principal strains of proximal bone structure in a cross-section of proximal fragment for the different screw configurations (Figure 8). The strain nephograms is based on the hypothesis that CCS cutout from femoral head may take place as a result of high strains in the weak area of the proximal bone fragment [20]. Areas characterized by strains larger than a cutout value of 0.9% maximum principal strain were assigned orange color to emphasize the regions in which the bone structure was vulnerable to yielding [21]. Model of inverted triangle showed the lowest volume of yielding strain in proximal cancellous bone structure indicating that the CCSs configuration of inverted triangle was less likely to be cutout (Figure 9).

### 3.3. Stress Distribution around Screw Holes

**Figure 10:**
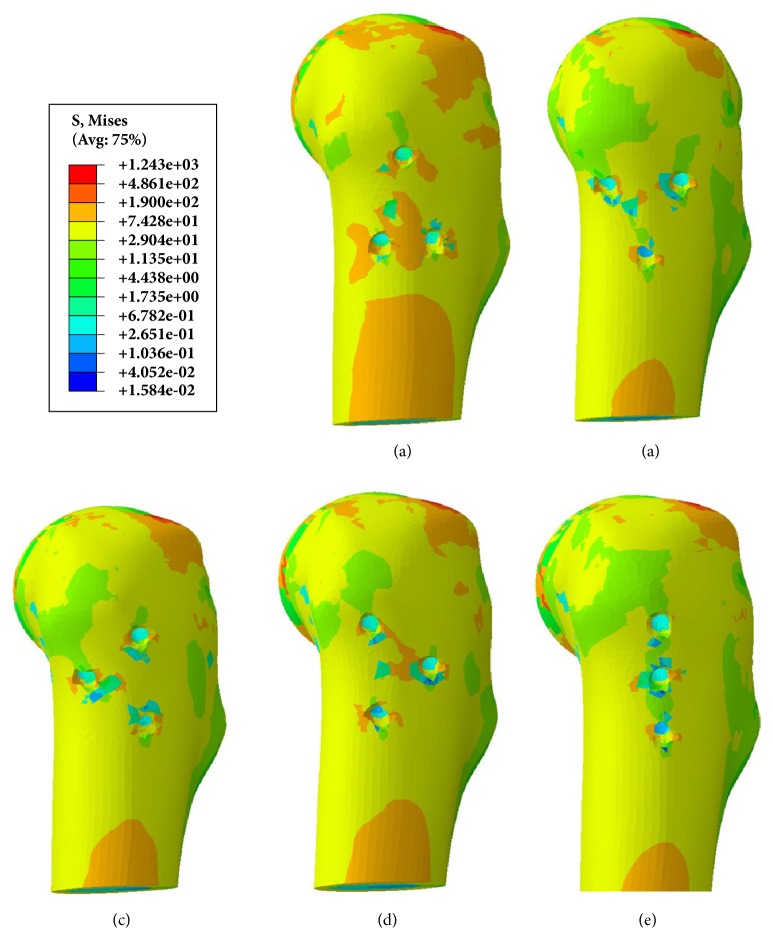
Stress distribution around the holes corresponding to fixation protocol after screws removal. (a) Triangle model. (b) Inverted triangle model. (c) Anterior triangle model. (d) Posterior triangle model. (e) Vertical model.

A comparison of the stress distribution around the holes for the five fixation protocols after screws removal was shown in Figure 10. The area of higher stress around the holes was largest after triangle screws removal when compared with other four models. In the scenario with triangle screws removal, the inferior two holes negatively affected the mechanical performance of the femur, and complication like subtrochanteric fractures may tend to occur.

## 4. Discussion

In the present study, we investigated whether different three-CCS configurations had the mechanical distinction for the fixation of unstable FNF and analyzed the effect of different configurations after screws removal on the strength of femur using FEA. This study demonstrated that screws of inverted triangle configuration underwent highest stress value under the increasing loads, and the bone structure showed the minimum yielding strains when compared to other configurations. The fixation strategy of triangle configuration was least recommended if patients were willing to remove the implants.

Some studies have evaluated the outcome of different screw types for the fixation of FNFs [22, 23], but without finding the statistical difference in clinical outcomes. Selvan et al. [7] demonstrated that triangular configurations had biomechanical advantage for the fixation of Pauwels type III fractures using synthetic bone models. But mechanical tests performed by Crowell et al. [24] and Benterud et al. [25] concluded that configuration of inverted triangle and diamond patterns provides better fixation of femoral head. And Yang et al. [8] reported that the inverted triangle configuration of screws increased the union rate when compared with triangle configuration. The FEA results in this study may provide some guidance in the clinical practice. The Figures 5 and 6 illustrated that inverted triangle screws underwent higher amount of stress than other configurations. This can be explicated by the condition that CCSs of inverted triangle provide a better anchorage than others, thereby displacing the stresses transmitted by the body weight, also demonstrated in Figures 8 and 9. The maximum principal strains and corresponding yield strain were used to evaluate the risk of screw cutout. Screw stability within the head is up to the adequate anchoring force in bone structure. It is necessary to avoid the condition that the bone is beyond its yield straining. Figure 9 showed the volume of bone susceptible to yielding in proximal bone structure, which indicated that the CCSs configuration of inverted triangle was less likely to be cutout.

In FNFs, CCSs configuration of inverted triangle showed mechanical and clinical advantage compared with other configurations. This may associate with three aspects. First, the two most distal screws of triangle configuration inevitably pass through the Ward triangle within the femoral neck, which leads to the decrease of bone density because of the absence of trabeculation [26]. Second, the dense trabeculae on the central and superior portion of the femoral head could provide greater bone anchorage [24, 25]. The two screws of inverted triangle configuration inserted into the dense trabeculae area exhibit greater holding strength than the only one screw of triangle configuration. Third, drilling twice on the less area of inferior portion induces the stress concentration on the subtrochanteric area, which may increase the prevalence of subsequent subtrochanteric fractures.

Zielinski et al. [27] concluded that implant removal after internal fixation of a FNF positively influenced quality of life, especially for those who were younger and more ambulatory and more often had a Pauwels III fracture and an evident implant back-out. But subtrochanteric fractures have been reported as a serious complication following removal of screws for FNF or slipped capital femoral epiphysis [11–13]. In this study, the results of FEA research showed that different screws' configurations displayed different stress distribution on the proximal femur. As shown in Figure 10, triangular configuration showed larger stress distribution area around the screw holes, indicating that triangle construct inclined to second fracture around the subtrochanteric area.

In this study, there is no experimental validation being performed, which definitely is a limitation. Nevertheless, our objective was to show the tendency rather than the absolute value of parameters. In this way, the lack of experimental validation is rationalized. Previous experimentally validated studies [15–18] employed the same loading and boundary conditions as this study. And it was just a static simulated biomechanical study and further biomechanical researches are needed to explore the cyclic loading conditions. Even so, this is the first FEA research comparing the mechanical distinction of five CCS configurations and the stress distribution around the holes corresponding to fixation protocol after screws removal. Besides, we have simulated the threaded tunnels left by CCS in each model and refined the mesh around the areas to make the parameter level more realistic.

## 5. Conclusions

In conclusion, using FEA modeling, we systematically evaluated the mechanical distinction in unstable femoral neck fracture models by varying CCS configurations and the stress distribution around the holes corresponding to screw configurations after fracture union. Our study indicated that different configurations for the unstable femoral neck fractures showed the different mechanical efficiency. Inverted triangle configuration showed the mechanical advantage and being less likely to be cutout. The fixation strategy of triangle configuration was least recommended if patients tended to remove the implants.

## Figures and Tables

**Table 1:** Distance from the surface of the femoral head to the tip of each screw in different models.

	Triangle			Inverted triangle			Anterior triangle			Posterior triangle			Vertical model		
	S	IP	IA	SP	SA	I	S	A	I	S	P	I	S	M	I

Distance(mm)	9.8	9.2	9.6	10.7	9.6	10.4	8.4	10.7	9.4	8.6	11.2	9.5	8.2	11.6	10.7

Abbreviation: S, superior; IP, inferoposterior; IA, inferoanterior; SP, superoposterior; SA, superoanterior; I, inferior; A, anterior; P, posterior; M, middle.

**Table 2:** Distance between screws in different models.

	Triangle			Inverted triangle			Anterior triagle			Posterior triangle			Vertical model	
	S-IP	IP-IA	IA-S	SP-SA	SA-I	I-SP	S-A	A-I	I-S	S-P	P-I	I-S	S-M	M-I

Distance(mm)	22.7	12.7	22.6	17.6	20.7	21.4	17.3	18.4	22.5	17.5	18.5	22.8	13.6	14.2

**Table 3:** Material properties of models in this study.

Ti-6AL-4V		Cortical bone		Cancellous bone	
E (GPa)	Poisson's ratio	E (GPa)	Poisson's ratio	E (GPa)	Poisson's ratio
105	0.35	16.8	0.3	0.84	0.2

**Table 4:** Parameters of the constitutive law.

		Triangle	Inverted triangle	Anterior triangle	Posterior triangle	Vertical model
Femur	Elements	788416	742684	777827	752670	759762
	Nodes	161809	152348	159510	154261	155471
	Mesh size	maximum: 3mm; minimum: 0.5mm				
Screws	Elements	56737	55998	55655	56435	57067
	Nodes	101995	100341	99884	100885	101736
	Mesh size	1mm				

**Table 5:** Maximum stress (Mpa) on each CCS of five models.

	Triangle			Inverted triangle			Anterior triangle			Posterior triangle			Vertical model		
	S	IP	IA	SP	SA	I	S	A	I	S	P	I	S	M	I
500N	224	197	212	260	276	225	218	207	204	257	182	223	217	186	217
1000N	444	400	426	524	543	451	437	416	419	511	372	454	432	368	441
1500N	659	608	643	797	803	675	657	625	642	759	568	692	643	543	670
2100N	865	819	860	1068	1068	899	872	837	872	990	768	934	846	709	902

## Data Availability

The data used to support the findings of this study are available from the corresponding author upon request.
